# Osteomyelitis Caused by Ralstonia mannitolilytica, a Rare Opportunistic Pathogen

**DOI:** 10.7759/cureus.24151

**Published:** 2022-04-14

**Authors:** Eftychios Papagrigorakis, Michail Vavourakis, Christos Vlachos, Dimitrios Zachariou, Athanasios Galanis, Vasileios Marougklianis, Vasileios Polyzois, Spiros Pneumaticos

**Affiliations:** 1 3rd Orthopaedic Department, KAT General Hospital of Attica, National and Kapodistrian University of Athens School of Medicine, Athens, GRC; 2 4th Orthopaedic Department, KAT General Hospital of Attica, Athens, GRC

**Keywords:** mannitolilytica, 16s ribosomal dna sequencing, bone transport, osteomyelitis, ralstonia

## Abstract

*Ralstonia* spp. are non-fermenting aerobic gram-negative rods found in humid environments, whose role as opportunistic human pathogens has lately been recognized. *Ralstonia mannitolilytica* is one of the three members of the *Ralstonia* genus (together with *Ralstonia pickettii* and *Ralstonia insidiosa*). Bone infections by *Ralstonia* spp. are very rare. We report a case of femoral osteomyelitis caused by *R. mannitolilytica*. Among literature search, only eight cases of bone infection due to the *Ralstonia* genus have been described, in all of which the causative agent was identified as *R. pickettii*. To our knowledge, this is the first reported case of osteomyelitis attributed to *R. mannitolilytica*. Despite its low virulence, *Ralstonia* has specific characteristics that promote its spread and shows high antibiotic resistance, partly due to its ability to create a biofilm. Identification of *Ralstonia* spp. poses unique difficulties as the distinction between the species of the genus is not straightforward. Additionally, the bacteria may be misidentified as other closely related species. Recent data suggests that modern spectrometry and gene sequencing techniques are essential to avoid these pitfalls. Susceptibility data about the genus is limited and based on a small number of case reports, therefore there is no standardized antibiotic susceptibility testing and European Committee on Antimicrobial Susceptibility Testing (EUCAST) breakpoints exist. The report aims is to provide useful information on the antibiotic selection and treatment suggestions to be followed for bone infections caused by the *Ralstonia* genus, along with a review on the literature of this emerging opportunistic pathogen.

## Introduction

*Ralstonia* spp. are non-fermenting aerobic gram-negative rods found in humid environments such as water, soil and plants [1]. *Ralstonia mannitolilytica*, formerly known as *Pseudomonas thomasii*, is one of the three members of the *Ralstonia* genus (together with *Ralstonia pickettii* and *Ralstonia insidiosa*) that have been lately recognized for their role as opportunistic human pathogens. Most reports in the literature for the *Ralstonia* spp. involve *R. pickettii*, which is the most clinically prevalent member of the genus. However, during the last 10 years, a few single case reports and hospital outbreaks have been attributed to *R. mannitolilytica* infection. These cases involve mainly immunocompromised patients, patients on hemodialysis or with respiratory problems. Most of them are associated with the use of contaminated solutions, including saline solutions, water for injections and disinfectants [1,2]. Despite its low virulence, *Ralstonia* shows high resistance to antibiotics, partly due to its ability to create biofilm, while its laboratory identification is trivial making it a difficult-to-treat microorganism [3].

We report a case of osteomyelitis of the femur caused by* R. mannitolilytica* in a young male patient after a motorcycle crash that led to an open femoral fracture. In current literature, only a few cases of bone infections caused by this genus are reported, mainly by *R. pickettii*, and none by *R. mannitolilytica*. This report aims to provide useful information for clinicians on the antibiotic selection and treatment of bone infections caused by the *Ralstonia* genus and a review of the literature.

## Case presentation

**Figure 1:**
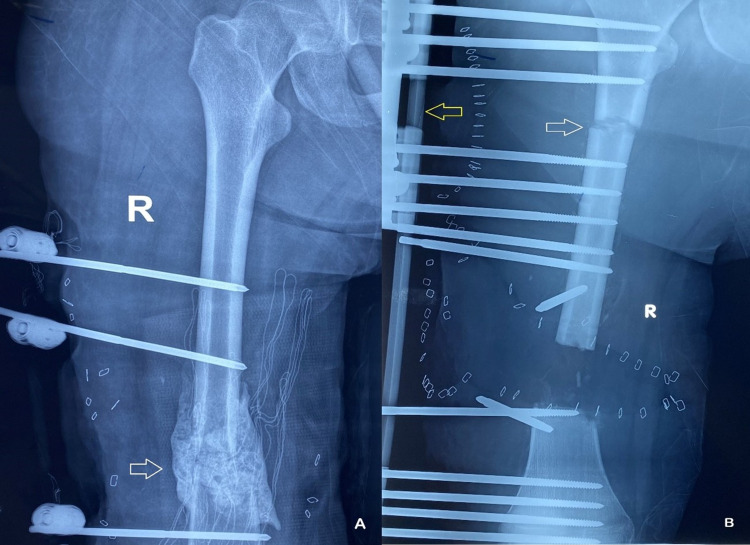
A. Sequential debridements resulted in a bone defect that was initially filled with polymethylmethacrylate (PMMA) cement (white arrow). B. The cement was later removed and a segmental bone transport was initiated to fill the bone defect and to correct the leg length discrepancy. The distraction site is seen proximally (white arrow) and the unilateral external fixator to the left (yellow arrow).

An 18-year-old male sustained an open diaphyseal fracture (Gustilo IIIb) on his right femur after a motorcycle accident. The accident took place off-road and the wound was massively contaminated with soil. The patient was set on an empirical combination of antibiotics (cefuroxime and amikacin). An extensive lavage and surgical debridement in the operation room followed, and the fracture was stabilized with external fixation. Postoperatively, the wound showed signs of infection. Sequential surgical debridements were deemed necessary (every second day for two weeks). Due to persistent inflammation the patient was operated on again, where bone and soft tissues were removed from the fracture site, leading to a 4 cm defect. The gap was filled with the interposition of gentamycin polymethylmethacrylate (PMMA) cement (Palacos®+G*, 0.5g gentamycin per 41.1g PMMA; Heraeus Kulzer GmbH, Hanau, Germany) (Figure 1A). Adequate bone specimens were sent to the laboratory for cultures (bacterial and fungal) and further analysis. After two complementary surgical debridements, the patient was reoperated to correct limb shortening with distraction osteogenesis technique. Proximal femoral corticotomy was performed (distraction site) and an external fixator (LRS; Orthofix, Milan, Italy) was used for the transport of the bone segment (Figure 1B). At the fracture site, the PMMA cement was removed and debridement to healthy bone margins with further removal of 3 cm of necrotic bone was performed (paprika sign). A split-thickness skin autograft from the left thigh was used to cover the skin deficit. The bone transport was initiated one week postoperatively, at an elongation rate of 1 mm per day to correct a total of 7 cm length discrepancy.

During the above surgical procedures, adequate bone specimens were obtained and sent for cultures. Specimens were plated onto 5% sheep blood agar, chocolate agar and MacConkey agar. The plates were incubated at 35-37 °C in ambient air. Plates were inspected for growth at 24 h and again at 48 h. The cultured colonies were identified as *R. mannitolilytica* by using the VITEK 2 automated identification system (bioMérieux, Marcy-l'Étoile, France). After testing positive for *Ralstonia* infection, further laboratory analysis by 16s ribosomal deoxyribose nucleic acid (rDNA) gene sequencing was performed for confirmation. A polymerase chain reaction (PCR) product was purified by using the Promega Wizard PCR Preps kit (Promega, Madison, WI, USA) according to the manufacturer’s instructions. Sequence analysis was performed with an Applied Biosystems 3700 DNA sequencer and the protocols of the manufacturer (PE Applied Biosystems, Foster City, CA, USA) by using the BigDye Terminator Cycle Sequencing Ready Reaction kit. The sequences were compared to sequences available in the GenBank database by using FASTA3, and the results obtained with FASTA3 were used to identify isolates to the species *R. mannitolilytica*. Antimicrobial susceptibility was performed by the broth microdilution method (MicroScanWalkAway plus; Beckman Coulter, Milan, Italy). As there are no European Committee on Antimicrobial Susceptibility Testing (EUCAST) susceptibility breakpoints available for *Ralstonia* spp., minimum inhibitory concentration (MIC) results were interpreted using the criteria used for *Pseudomonas spp*. and *Acinetobacter spp*. for trimethoprim-sulfamethoxazole.

After the identification of the *Ralstonia* spp. and the antibiotic susceptibility testing, the initial regimen was discontinued and replaced by tigecycline (Table 1). The antibiotic was administered intravenously for a total of three months. Capsofungin was also administered for two months to treat a *Candida parapsilosis* superinfection.

**Table 1:** Susceptibility testing of presented case. MIC: minimum inhibitory concentration

Drug	MIC	Interpretation
Amikacin	> 32	R
Ampicillin/Sulbactam	> 16/8	R
Ampicillin	> 16	R
Cefepime	> 16	R
Cefotaxime	> 32	R
Cefoxitin	> 8	R
Ceftazidime	> 16	R
Cefuroxime	> 16	R
Ciprofloxacin	> 2	R
Ertapenem	> 1	R
Fosfomycin	> 64	R
Gentamycin	> 8	R
Imipenem	> 8	R
Levofloxacin	> 4	R
Meropenem	> 8	R
Moxifloxacin	> 1	R
Nalidixic Acid	> 16	R
Nitrofurantoin	> 64	R
Piperacillin/Tazobactam	> 64	R
Piperacillin	> 64	R
Tetracycline	> 8	R
Tigecycline	≤ 2	S
Tobramycin	> 8	R
Trimethoprim/Sulfamethoxazole	> 4/76	R
S = Susceptible I = Intermediate R = Resistant MIC = mcg/ml (mg/L)		

**Figure 2:**
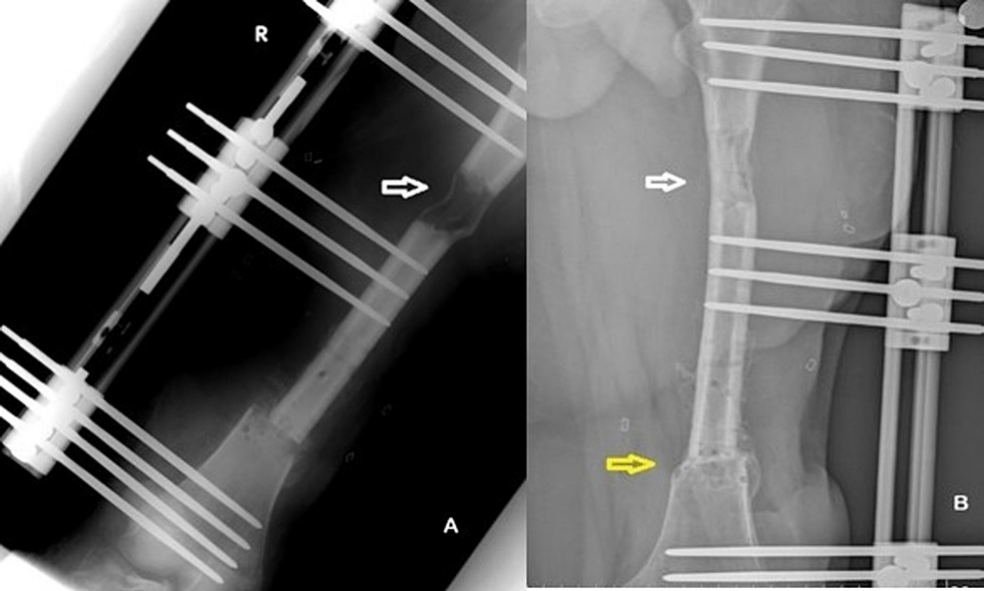
A. End of bone transport at three months post-operatively. New bone formation is obvious at the distraction site (white arrow) B. Consolidation at the distraction site (white arrow) and callus formation at the docking site (yellow arrow) eight months postoperatively.

**Figure 3:**
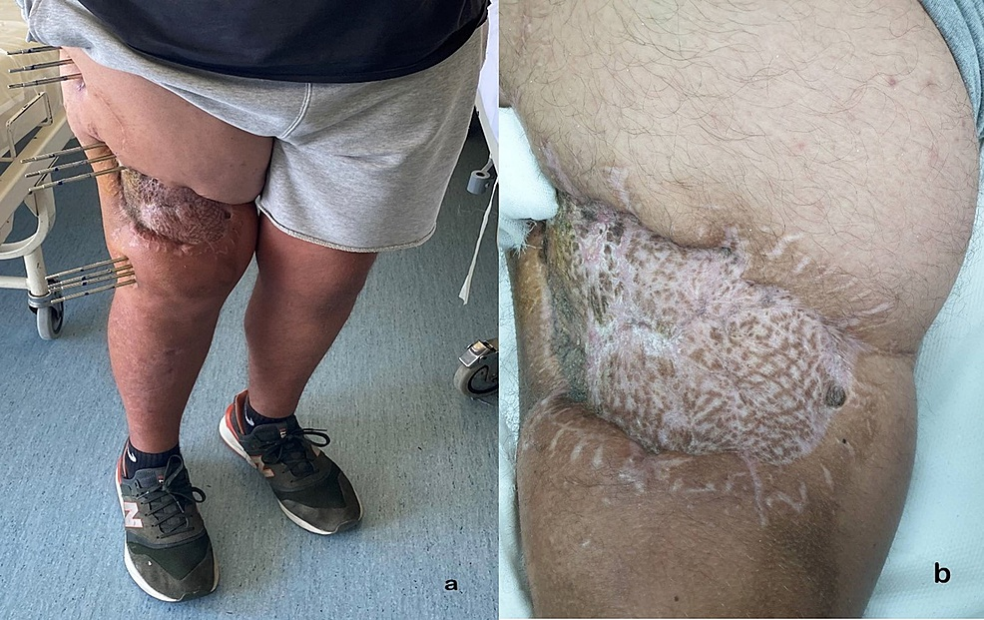
A. Full weight-bearing after the removal of external fixation device. B. skin autograft

The postoperative course of the patient was uncomplicated. Two months after the start of the bone transport, partial weight-bearing was allowed and the patient was able to ambulate with the use of crutches. The elongation process lasted for 85 days. Bone healing was uneventful and no further procedures were necessary for consolidation at the docking site (Figure 2). The external fixation device was removed 10 months postoperatively. The bone transfer was successful with evident callus formation from the third month, while the leg length discrepancy was eliminated. On his last visit, one year after the last operation, the patient is ambulatory with a functional right lower limb and a minor decrease in the hip and knee range of motion (Figure 3). The range of motion (or ROM) of the hip is 0-100˚ of flexion and of the knee is 0-90˚. The patient had a 96/100 Harris hip score (HHS) and a 90/100 Knee Outcome Survey-Activities of Daily Living Scale (KOS-ADLS) rating. He complains of mild pain while the inflammatory markers on his laboratory blood tests are negative.

## Discussion

*R. mannitolilytica* is emerging as an opportunistic human pathogen, mostly implicated in hospital-acquired infections [1]. Although *R. mannitolilytica* presence in humid environments is common, bone infections are rarely reported. Most cases in literature are nosocomial outbreaks in immunocompromised patients, patients on hemodialysis or with malignancies, and premature infants [2,4-9]. Special attention has been drawn to patients with cystic fibrosis as *R. mannitolilytica* infection seems to worsen the prognosis of the disease [10,11]. The most common manifestation of the infection is bacteremia related to a central venous catheter that can lead to sepsis, meningitis or peritonitis [1,9].

Despite its low virulence, *R. mannitolilytica* species has specific characteristics that promote its spread. These microorganisms are capable of creating a polysaccharide matrix cover (biofilm), which enhances their resistance to antibiotics and helps them survive in harsh conditions for a prolonged time [12-14]. They can also penetrate 0.2 μm filters that are used for the terminal sterilization of most medical products [4,5]. They have been reported to survive mild hospital disinfectants like chlorohexidine 5% and ethacridine lactate solutions [5,8]. These factors contribute to the dissemination of the bacteria and the contamination of medical equipment (air conditioners) and products (normal saline and other solutions flushed through indwelling devices), resulting in the aforementioned hospital outbreaks [15].

Bone infections by the *Ralstonia* genus are very rare. Two cases of spondylitis, one case of osteomyelitis of the trapezium of the hand, one case of septic arthritis of the elbow and knee, and four cases of periprosthetic joint infection have been described in the literature so far. The basic features of these bone infections are summarized in Table 2. The causative bacteria in all cases were identified as *Ralstonia pickettii*. To our knowledge, this is the first reported case of a bone infection attributed to *Ralstonia mannitolilytica*. 

**Table 2:** Bone infection cases attributed to Ralstonia genus among current literature. HIV: human immunodeficiency virus, HCV: hepatitis C virus, DM: diabetes mellitus, THA: total hip arthroplasty

Author	age	Infection Site	Bacteria Isolated	Co-morbidities	Antibiotic Regimen	Therapy Duration	Result
Wertheim 1992 [16]	71 male	L4,L5 Spondylitis	R. pickettii	Hemodialysis, chronic renal failure, DM	Trimethoprim - sulfamethoxazole + discectomy, decompressive laminectomy	6w PO	Healed
Elsner 1998 [17]	40 female	T7,T9 spondylitis	R. pickettii, S. epidermidis, P. acnes	--	Ciprofloxacin	12w PO	Healed
Zellweger 2004 [18]	24 male	Right elbow, left knee septic arthritis	*R. pickettii*, group A *Streptococcus*	IV drug user, septicemia, meningitis, endocarditis	Ceftriaxone (no results after 2w IV), followed by ciprofloxacin with penicillin G + surgical drainage	2w PO	Septic arthritis resolved
DeGeorges 2005 [19]	29 male	Trapezium osteomyelitis	R. pickettii	HIV, HCV, hemodialysis	Ticarcillin - clavulanate (no results after 3m), amoxicillin - clavulanic acid + gentamycin, then sulfamethoxazole+ rifampicin + surgical removal of trapezium	3m PO	Healed
Birlutiu 2017 [20]	83 male	THA infection	R. pickettii, P. aeruginosa	Coronary artery disease, DM	Sulfamethoxazole + multiple stage exchange with antibiotic spacers and surgical debridements	12w PO	Healed

The biochemical identification of the *Ralstonia* genus poses special challenges for the microbiologist. The distinction between the species of the genus is not straightforward. Additionally, the bacteria may be misidentified as other closely related species with similar characteristics (mainly of the *Burkholderia cepacia* complex or *Pseudomonas* species) [3,9]. Key knowledge for the biochemical distinction between the species of the genus is that *R. mannitolilytica* metabolizes only mannitol but not nitrate or arabinose, *R. insidiosa* metabolizes nitrate but not mannitol or arabinose, *R. pickettii *metabolizes nitrate and arabinose but not mannitol [21]. Recent data suggest that modern spectrometry and gene sequencing techniques are important to avoid these pitfalls. 16s ribosomal DNA based assays are a sensitive and specific methodology for identification. In our case the VITEK 2 automated identification system yielded a rapid and accurate species-level identification and, in accordance with other studies, the result was confirmed using the 16S rDNA as the reference method [13,22]. A modern mass spectrometry method (Matrix assisted laser desorption ionization-time of flight - MALDI-TOF technique), on the other hand, is a rapid [12], reliable and low cost method that has contributed to the proper identification of these non-fermenting bacteria [23]. However, most researchers agree that the confirmation by 16s rDNA gene sequencing is the most accurate way to isolate and identify the microbia [3].

The *Ralstonia *genus seems to have developed resistance against many antibiotic agents. The treatment protocol needs careful planning as the genus produces various enzymes that can hydrolyze antibiotics and resistance to aminoglycosides and beta-lactams is frequently reported [3]. Special attention has been drawn to the rising resistance against many modern antibiotics such as ceftazidime, aztreonam and carbapenems [3,12]. In a recent study, Suzuki et al. identified a species-specific extended spectrum oxacillinase (OXA60) with carbapenem-hydrolyzing properties that contribute to the genus resistance against imipenem and carbapenem [24]. Co-trimoxazole and ciprofloxacin are generally considered effective against the genus, while also tigecycline has been shown to have good in vitro activity against *Ralstonia* spp. [25]. The drug susceptibility profile of the *Ralstonia* genus in bone infections found in literature is summarized in Table 3. In most cases the microorganisms were susceptible to many antibiotics. On the contrary, in our case the *R. mannitolilytica* presented with a multidrug resistance profile (Table 1). The selected combination was, at first, cefuroxime and amikacin, which proved to be ineffective. After the identification of the *R. mannitolilytica* and the susceptibility testing, tigecycline was the only effective antibiotic and was administered for three months. Despite the osteoblastic activity inhibitory potential of tetracyclines, the use of tigecycline was deemed necessary by our institution's infectiology committee for the eradication of the osteomyelitis. Due to the long duration of the treatment, the course was complicated by positive blood cultures of *Candida parapsilosis*, so caspofungin was added to the given regimen [3].

**Table 3:** Susceptibility profile of Ralstonia bone infections.

Author	Wertheim [16]	Elsner [17]	Zellweger [18]	Birlutiu [20]
Microorganism	R. pickettii	R. pickettii	R. pickettii	R. pickettii
Susceptible	Ampicillin, Ampicillin-Sulbactam, Piperacillin, Cephalothin, Cefoxitin, Trimethoprim-Sulfamethoxazole, Ciprofloxacin, Amoxicillin-Clavulanic acid, Cefuroxime, Ceftriaxone, Ceftazidime, Imipenem, Aztreonam, Ticarcillin-Clavulanic acid, Cefotaxime, Cefoperazone, Mezlocillin.	Ampicillin, Mezlocillin, Piperacillin, Cefoxitin, Cefotaxime, Tetracycline, Trimethoprim- Sulfamethoxazole, Ciprofloxacin.	Piperacillin, Piperacillin-Tazobactam, higher generation cephalosporins (including Cefuroxime, Ceftriaxone, Ceftazidime, Cefepime), Imipenem, Trimethoprim-Sulfamethoxazole, Ciprofloxacin.	Ticarcillin, Piperacillin, Cefepime, Imipenem, Meropenem, Ciprofloxacin, Pefloxacin, Minocycline, Cotrimoxazole.
Intermediate			Aztreonam, Netilmicin.	Ceftazidime
Resistant			Ampicillin-Clavulanate, Ticarcillin- Clavulanate, Meropenem, Gentamicin, Tobramycin, Amikacin.	Aztreonam, Amikacin, Gentamicin, Colistin.

It should be noted that the susceptibility data about the genus is limited and based on a small number of case reports, thus no standardized antibiotic susceptibility testing and EUCAST breakpoints exist for the laboratories [13]. In most bone infections caused by the *Ralstonia* genus, ciprofloxacin or co-trimoxazole was used as the definitive treatment as shown in Table 1, but in our case the isolated *Ralstonia* genus was found to be resistant to both. Multi-drug resistance is of great concern as these microorganisms act not only as human pathogens, but also as potential reservoirs of resistance genes in hospital settings [8].

## Conclusions

The increasing detection of *Ralstonia* spp. in hospital settings, along with the emergence of multi-resistant strains of *R. mannitolilytica*, represent a reason of concern, particularly in the case of vulnerable patients. Identification is not straightforward and special attention is essential with the use of modern techniques (16s rDNA gene sequencing and spectometry). Despite that *Ralstonia* spp. are usually not recognized as major pathogens, their multidrug resistance, biofilm formation potential and ability to survive in the environment are factors that should never be underestimated. Clinicians and microbiologists should pay attention to the potential of these opportunistic microorganisms being the cause of severe bloodstream infections.
